# Mecasin treatment in patients with amyotrophic lateral sclerosis: study protocol for a randomized controlled trial

**DOI:** 10.1186/s13063-018-2557-z

**Published:** 2018-04-13

**Authors:** Sungha Kim, Jae Kyoun Kim, Mi Ju Son, Dongwoung Kim, Bongkeun Song, Ilhong Son, Hyung Won Kang, Jongdeok Lee, Sungchul Kim

**Affiliations:** 1 Clinical Research Division, Korea Institute of Oriental Medicine, 1672 Yuseong-daero, Yuseong-gu, Daejeon, 34054 Republic of Korea; 2 Department of Global Public Health and Korean Medicine Management, Graduate School, Kyung Hee University, 26 Kyungheedae-ro, Dongdaemun-gu, Seoul, 02447 Republic of Korea; 3 Center of ALS/MND, Wonkwang University Gwangju Medical Hospital, 1140-23 Hyjae-ro, Nam-gu, Gwangju, 61729 Republic of Korea; 4 Department of Neurology, Wonkwang University Sanbon Hospital, 327 Sanbon-ro, Gunpo-si, Gyunggi-do 15865 Republic of Korea; 5 Department of Korean Neuropsychiatry Medicine, Wonkwang University Sanbon Hospital, 327 Sanbon-ro, Gunpo-si, Gyunggi-do 15865 Republic of Korea

**Keywords:** Motor neuron disease, Amyotrophic lateral sclerosis, KCHO-1, Mecasin, Randomized controlled trial, Protocol

## Abstract

**Background:**

Amyotrophic lateral sclerosis (ALS) is a fatal neurodegenerative disease that causes paralysis of limb, swallowing, and breathing muscles. Riluzole, the Food and Drug Administration-approved drug for ALS, provides minimal benefit, prolonging patient life by only 2–3 months. Previous studies have found a neuro-protective and anti-neuroinflammatory effect of Mecasin, with retrospective studies providing suggestive evidence for a beneficial effect of Mecasin. The aim of this study was to develop a protocol to determine the proper dosage of Mecasin.

**Methods:**

This is a phase II-A, multi-center, randomized study with three arms. Thirty-six patients with ALS will be randomly assigned to one of three groups, each receiving the standard treatment with 100 mg of riluzole in addition to one of 1.6 g of Mecasin, 2.4 g of Mecasin, or a placebo. The Primary outcome is the Korean version of the Amyotrophic Lateral Sclerosis Functional Rating Scale-Revised result after 12 weeks of treatment. Secondary outcomes include results of the Short Form Health Survey-8, Medical Research Council Scale, Visual Analogue Scale for Pain, Hamilton Rating Scale for Depression, Fatigue Severity Scale, Patient Global Impression of Change, pulmonary function test, forced expiratory volume in 1 s and its ratio to forced vital capacity, creatine kinase, and body weight. The frequencies of total adverse events and serious adverse events will be described and documented. The trial protocol has been approved by the Institutional Review Board of the Wonkwang University Gwangju and Sanbon Hospital (2016–5-4 and 2016–34-01, respectively). An Investigational New Drug status (30731) was granted by the Korea Food and Drug Administration.

**Discussion:**

This trial will aim to identify the optimal dosage of Mecasin. Additionally, it will test the efficacy and safety of Mecasin in conjunction with standard treatment, riluzole, for alleviating the functional decline in patients with ALS.

**Trial registration:**

Korean National Clinical Trial Registry CRIS; KCT0001984. Registered on 28 July 2016.

**Electronic supplementary material:**

The online version of this article (10.1186/s13063-018-2557-z) contains supplementary material, which is available to authorized users.

## Background

Amyotrophic lateral sclerosis (ALS) is a lethal neurodegenerative disorder characterized by focal weakness of the limbs or bulbar muscles, and progressing to involve all skeletal muscles and the loss of both upper and lower motor neurons [1]. Symptoms may rapidly progress over the course of as little as 2–4 years, although some patients may live for up to 10 or more years [2, 3]. Once paralysis of the respiratory muscles occurs, the patient can no longer survive without permanent ventilatory support.

Although various drugs are currently being developed to treat ALS, no drug with a definitive therapeutic effect is yet available. The current status of treatment for ALS includes riluzole and edaravone, which slow ALS progression, and nuedexta, which that reduces emotional outburst; however, none of these drugs is able to completely extend survival, but rather induce a slowing down of patient symptom deterioration [4].

Due to the unremarkable effects of conventional medical treatments, various complementary and alternative therapies are currently used in clinical settings [5]. We have previously prescribed the herbal drug*,* modified *jakyakgamchobuja-tang* to patients with ALS. A retrospective study showed that 3 months of combined therapy consisting of the herbal drug, acupuncture, pharmacopuncture, and needle-embedding therapy alleviated ALS progression. The Korean version of the Amyotrophic Lateral Sclerosis Functional Rating Scale-Revised (K-ALSFRS-R) score increased from 28.42 ± 7.83 to 29.08 ± 7.99 after 1 month of treatment, and decreased to 28.16 ± 8.23 after 3 months. However, it severely decreased to 21.33 ± 9.93 at 8 months from discharge [6]. Another study showed that 30 days of the combined therapy led to a K-ALSFRS-R score increase from 28.42 ± 7.83 to 29.08 ± 7.99 [7]. Mecasin, also known as KCHO-1, a herbal extract from modified *jakyakgamchobuja-tang*, has shown neuro-protective and anti-neuroinflammatory effects and safety in both in vitro and in vivo trials [8–14]. The aim of this study was to develop a protocol to assess the optimal dosage of Mecasin, and to test whether Mecasin alleviates the functional decline in patients with ALS. The study will be a phase II-A, randomized, multi-center, blinded, parallel-group trial with three groups. Additionally, it will aim to assess safety issues.

## Methods/Design

### Primary objective

The primary objective is to investigate the optimal dosage of Mecasin.

### Secondary objectives

The secondary objectives are to determine whether Mecasin, combined with standard ALS treatment, significantly slows the rate of functional deterioration in patients with ALS over 12 weeks, compared with standard treatment alone, and to monitor the safety of Mecasin combined with standard ALS treatment over 16 months. We also aimed to determine whether this treatment affects health-related quality of life, depression, muscle strength, pain, fatigue, pulmonary function, creatine kinase, or body weight in patients with ALS over 12 months.

### Design and setting

**Fig. 1:**
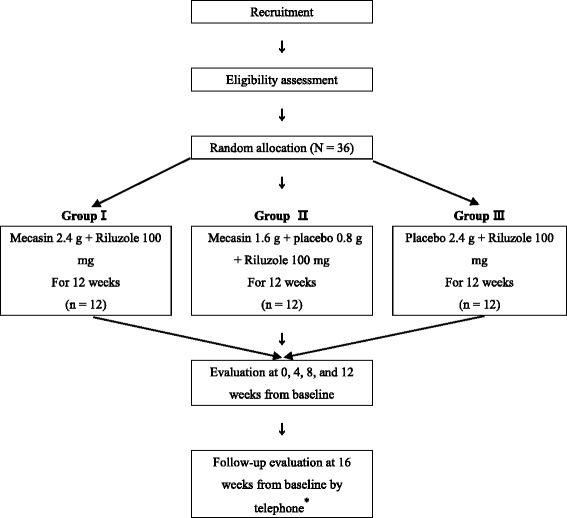
Flow diagram of the study participants. *Follow-up evaluations for adverse events will be conducted by a Clinical Research Coordinator

**Fig. 2:**
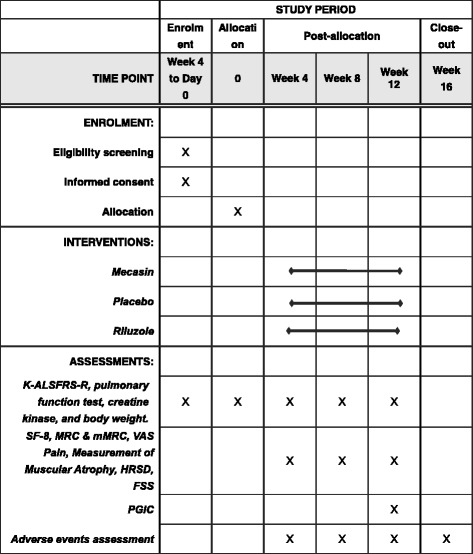
The SPIRIT trial schedule of enrolment, intervention, and assessment. *K-ALSFRS-R* Amyotrophic Lateral Sclerosis Functional Rating Scale-Revised, *SF-8* Short Form Health Survey 8, *MRC* Medical Research Council scale for muscle strength, *mMRC* Modified Medical Research Council Scale for Dyspnoea, *VAS* Pain Visual Analogue Scale for Pain, *HRSD* Hamilton Rating Scale for Depression, *FSS* Fatigue Severity Scale, *PGIC* Patient Global Impression of Change

This will be a phase II-A, multi-center, randomized, double-blind, placebo-controlled trial. All patients will have taken riluzole for more than 3 months before screening and will be committed to take riluzole during trials. The trial will be conducted at South Korea Wonkwang University Gwangju Hospital and Sanbon Hospital. The flow diagram of the trial is shown in Fig. 1. We will test the effects of 1.6 g and 2.4 g of Mecasin to investigate optimal dosage and treatment efficacy. The schedule of study procedures is depicted in Fig. 2 and details are provided in Table 1.

**Table 1:** Schedule of study procedures

	Screening	Treatment and evaluation				
		Baseline			Final	
Visit	Visit 1	Visit 2	Visit 3	Visit 4	Visit 5	Follow-up
Schedule	Week −4 to Day 0	Week 0	Week 4	Week 8	Week 12	Week 16
Informed consent	✓					
Demographic characteristics	✓					
Medical history	✓					
Inclusion/Exclusion criteria	✓					
Chest x-ray	✓					
Pregnancy test	✓				✓	
Electrocardiogram	✓				✓	
Randomization		✓				
Medications	✓	✓	✓	✓	✓	
Vital sign	✓	✓	✓	✓	✓	
Body weight	✓	✓	✓	✓	✓	
Pulmonary function test (FEV_1_/FVC)	✓	✓	✓	✓	✓	
Blood tests^a^	✓	✓	✓	✓	✓	
K-ALSFRS-R	✓	✓	✓	✓	✓	
Short Form Health Survey 8		✓	✓	✓	✓	
Medical Research Council Scale for muscle strength		✓	✓	✓	✓	
Visual Analogue Scale for Pain		✓	✓	✓	✓	
Measurement of Muscular Atrophy		✓	✓	✓	✓	
Hamilton Rating Scale for Depression		✓	✓	✓	✓	
Fatigue Severity Scale		✓	✓	✓	✓	
O_2_ saturation and CO_2_ pressure test		✓	✓	✓	✓	
Provision/collection of test medication and placebo		✓	✓	✓	✓	
Adverse events assessment			✓	✓	✓	✓
Patient Global Impression of Change					✓	

^a^Blood (5 mL) will be collected from each patient to measure creatine kinase and other markers

*K-ALSFRS-R* Amyotrophic Lateral Sclerosis Functional Rating Scale-Revised

### Participants

#### Inclusion criteria

The inclusion criteria are (1) men and women between the ages of 20 and 80 years; (2) a diagnosis of ‘laboratory supported probable’ , ‘probable’ or ‘definite’ ALS according to the revised El Escorial criteria [15]; (3) an K-ALSFRS-R score greater than 20; (4) treatment with 100 mg of riluzole up to 3 months prior to screening; (5) the ability to visit the hospital alone or with the assistance of a caregiver; and (6) the ability to provide voluntary written informed consent.

#### Exclusion criteria

The exclusion criteria are (1) patients with ≤ 30% forced vital capacity; (2) patients with other neurological diseases, including fronto-temporal dementia with communication difficulties and Parkinson’s disease; (3) patients with cardiovascular disease, including ischemic heart disease; (4) patients with hepatic diseases such as hepatic cirrhosis, hepatic cancer and active hepatitis, or aspartate transaminase/alanine transaminase greater than three times the upper normal limit; (5) patients with cholecystitis or biliary obstruction; (6) patients with renal failure or undergoing renal dialysis; (7) patients who have had liver or kidney transplants; (8) patients with interstitial lung disease, as observed on a chest x-ray; (9) patients who have experienced post-operative complications; (10) patients with a history of hypersensitivity to any component of the experimental medications or a similar medication class; (11) patients with genetic disorders (Kennedy’s disease, spinal muscular atrophy type 4) or diagnosis of motor neuron disease due to heavy metal poisoning; (12) patients who have received other experimental medications/procedures within 4 weeks of participating in this trial; (13) pregnant women, lactating women, and women of childbearing age who plan on becoming pregnant (i.e., no history of hysterectomy, bilateral tubal ligation, or bilateral oophorectomy within 2 years of menopause) and who do not agree to use medically appropriate contraceptives (e.g., oral medication, hormone implants, intrauterine devices, condoms, or spermicides), or men who do not agree to use appropriate contraceptives with female partners; (14) patients with primary lateral sclerosis and experiencing only upper motor neuron symptoms or with progressive muscular atrophy and experiencing only lower motor neuron symptoms; (15) patients with bleeding diathesis; (16) patients with malignant tumors; (17) patients who have undergone mechanical ventilation or tracheostomy and percutaneous endoscopic gastrostomy; (18) patients with active viral infections (hepatitis B virus antigen, hepatitis C virus antibody, human immunodeficiency virus antibody, cytomegalovirus immunoglobulin M, Epstein–Barr virus immunoglobulin M, herpes simplex virus type 2 immunoglobulin M, syphilis); (19) patients with a conduction block, including chronic inflammatory demyelinating polyneuropathy and multiple sclerosis; and (20) patients deemed by a clinical research investigator to be inappropriate for participation in the clinical trial.

#### Participant recruitment

The present study aims to recruit 36 participants; all participants will provide written informed consent prior to participation in the study. The participants will be recruited via an advertisement flier and the Internet.

#### Criteria for discontinuing the allocated interventions

The withdrawal criteria are (1) withdrawal of consent by patients or their agents; (2) use of the medication no longer possible due to adverse events or abnormal findings on laboratory tests; (3) an overall compliance rate of less than 80% during the medication period; (4) a violation of the inclusion/exclusion criteria confirmed during the clinical trial; (5) use of combination medication unavoidable; (6) impossible follow-up; and (7) judgment that the trial is difficult to continue by any investigator.

### Sample size

The primary purpose of this trial is to investigate the appropriate dosage for Mecasin, and to examine the efficacy and safety of Mecasin combined with the standard treatment, riluzole. Considering a dropout rate of 20%, 12 participants will be assigned to each of the three groups.

### Randomization, allocation concealment, and blinding

The randomization codes will be generated by an independent statistician with the ratio of Mecasin 1.6 g/day, Mecasin 2.4 g/day, and placebo treatments as 1:1:1 using SAS (Version 9.3, SAS institute. Inc., Cary, NC, USA). The code will be written on a piece of paper, put in a sealed envelope, and stored in a double-locked cabinet. After eligible participants have signed an informed consent form, a researcher who is not involved in recruitment or assessment will open the envelopes and assign the randomization codes. The opened envelope will be stored separately in a double-locked cabinet. Mecasin and placebo will be manufactured as identical-looking tablets. The tablets will be packaged and labelled by a manufacturer who is not involved in participant recruitment, assessment, or assignment. Allocation concealment will be maintained throughout the trial.

### Intervention

#### Mecasin

Mecasin, also known as KCHO-1, contains 30% ethanol extracts of nine medicinal herbs, including *Curcuma longa*, *Salvia miltiorrhiza*, *Gastrodia elata*, *Chaenomeles sinensis*, *Polygala tenuifolia*, *Paeonia lactiflora*, *Glycyrrhiza uralensis*, *Atractylodes lancea*, and processed *Aconitum carmichaeli*. Mecasin will be manufactured by Hanpoong Pharm and Foods Co. (Chonju, South Korea) using certified Good Manufacturing Practices by the Ministry of Food and Drug Safety, and produced as a rectangular maroon tablet with a film coating.

Results of single toxicity tests have revealed a no-observed-adverse-effect level at > 5000 mg/kg/day, while animal studies have reported efficacy at doses of between 250 and 500 mg/kg/day [10, 11]. When the optimum dose is set based on this range, the effective dose for a 60-kg adult is between 15 and 30 g/day, with safe doses up to 120 g/day. The safe dose for this trial has been calculated as 5000 mg/kg × (6/37) × (1/10) × 60 kg = 4.8625 g, while the effective dose has been calculated as 250–500 mg/kg × (3/37) × 60 kg = 1.216–2.432 g. Dosages for each group were determined by selecting a safe dose of 1.6 g (low-dose group) and a safe/effective dose of 2.4 g (middle-dose group).

#### Placebo

The placebo will be a rectangular maroon tablet with a film coating manufactured by the same pharmaceutical company that will manufacture Mecasin. The pharmaceutical ingredients of the placebo tablet will include microcrystalline cellulose, corn starch, light anhydrous silicic acid, carboxymethylcellulose calcium, caramel color, magnesium stearate, a coating substance (hypromellose 2910, polyethylene glycol 6000), and a color additive (black iron oxide, red iron oxide).

#### Riluzole

Riluzole (2-amino-6-trifluoromethoxybenzothiazole, Rilutek®) is currently the standard treatment of ALS. All participants will take riluzole at 100 mg/day.

### Primary outcome

The primary outcome is the difference in the total score on the K-ALSFRS-R from baseline to the last evaluation after the 12th week of treatment. The K-ALSFRS-R [16] is a reliable and useful scale for the evaluation of functional status in patients with ALS. The scale contains 12 questions to assess gross motor tasks, fine motor tasks, bulbar function, and breathing function [17]. A blinded trained researcher will assess the scale. If possible, the same researcher will perform the evaluation at all patient visits.

### Secondary outcomes

The secondary outcomes are the degree of variation of scores at baseline to the last evaluation at the 12th week of treatment of the following measures: the Short Form Health Survey 8 (SF-8), Medical Research Council (MRC) scale for muscle strength, Visual Analogue Scale for Pain (VAS Pain), measurement of muscular atrophy, Hamilton Rating Scale for Depression (HRSD), Fatigue Severity Scale (FSS), Patient Global Impression of Change (PGIC), pulmonary function test, creatine kinase, and body weight.

#### SF-8

The SF-8, a brief version of the widely used SF-36, is a health investigation questionnaire consisting of eight questions that will be administered by a clinical coordinator to assess patients’ physical and mental quality of life, an important dimension when caring for people with ALS [17].

#### MRC and modified MRC (mMRC) for Dyspnea scale

The MRC scale is a five-grade scale used to evaluate muscle strength of both upper and lower extremities [17], and the mMRC for Dyspnea scale is a four-grade scale to evaluate the effects of breathlessness on mobility [18].

#### VAS Pain

As more than 70% of patients with ALS complain of physical pain [19], the VAS Pain is a self-rated scale that will be used to provide a more objective description of the subjective pain experienced in the head and neck, shoulders, upper radicular group, lower back, and lower extremities.

#### Measurement of muscular atrophy

Upper extremity muscle loss will be assessed by measuring the circumference of the arm at the midpoint between the tip of olecranon process and the tip of acromion process. Lower extremity muscle loss will be assessed by measuring the circumference at 15 cm above the upper edge of the bilateral patella [20, 21].

#### HRSD

As patients with ALS complain of depression as the disease progresses [22], the HRSD will be used to evaluate the severity of depression symptoms. The questionnaire consists of 68 items divided into four categories of 17 items each.

#### FSS

Fatigue is a common symptom in ALS [23]. The FSS is used to evaluate fatigue in patients with ALS and consists of nine items, each graded on a 7-point scale.

#### PGIC

The PGIC is a self-reported questionnaire used to evaluate the severity of symptoms, therapeutic responses, and changes in the therapeutic effects during treatment. Patients evaluate overall improvement in symptoms on a 7-point scale [24].

#### Pulmonary function test, creatine kinase, and body weight

ALS is a motor neuron disease resulting in the loss of muscular strength and eventual paralysis of the respiratory muscles. We will measure pulmonary function using spirometry (Spirolab III, Medical International Research), forced expiratory volume in 1 s, and its ratio to forced vital capacity. To evaluate the increase in the levels of creatine kinase due to muscle damage [25], creatine kinase measurement will be included in blood tests. As progression of muscular atrophy could affect body weight, body weight will also be measured.

### Statistical analysis

#### Primary analysis

The population used in the analyses will be the intention-to-treat population. Analyses of covariance (ANCOVA) will be performed to evaluate differences in K-ALSFRS-R scores among the groups at week 12 compared to baseline. The covariates for the ANCOVA will include age, sex, K-ALSFRS-R score, pulmonary function test score, creatine phosphokinase level, body weight, muscle circumference, bulbar onset, and the survey scores used in the secondary outcomes. Moreover, differences before and after treatment within each treatment group will be tested and compared using paired *t*-tests or Wilcoxon signed-rank tests. A repeated-measures analysis of variance will be performed to evaluate changes per visit in each group.

#### Secondary analysis

An ANCOVA will be used to compare the changes of secondary outcomes after 12 weeks of treatment among groups. Paired *t*-tests will be used to analyze internal variation of the results obtained at week 12 compared to those obtained at baseline in each group. A repeated-measures analysis of variance will be performed to analyze changes per visit in each group.

### Adverse events

All undesirable medical findings that have not been observed prior to the clinical trial will be classified as adverse events. All unexpected responses related to Mecasin and riluzole will be recorded. If serious adverse events occur, these will be notified to the sponsor, Hanpoong Pharm and Foods Co., the Institutional Review Board (IRB), and the Ministry of Food and Drug Safety. The number of adverse events in the two Mecasin groups will be compared using the χ^2^ or Fisher’s exact tests.

### Monitoring

Regular monitoring will be conducted by HC & Management Inc. independently from the study sponsor to control data quality. They will confirm whether the record of the case report form is accurate by comparing it to the source and whether the practical procedures of the trial follow the approved protocol.

### Ethics

The IRBs of the Korean Medicine Hospital of Wonkwang Gwangju and of Sanbon University have approved the protocol (IRB approval numbers 2016-5-4 and 2016-34-01, respectively). This study is registered with the national clinical trial registry Clinical Research Information Service, which is a primary registry of the World Health Organization International Clinical Trials Registry Platform (KCT0001984). An Investigational New Drug status (30731) was granted by the Korea Food and Drug Administration. Any protocol modifications will be approved by the IRBs and the Korea Food and Drug Administration prior to implementation.

## Discussion

There are large variations in the reported annual incidence of ALS in Europe, Asia, and North America, ranging from 2.1 to 8.5 per 100,000 population [26]. There is currently no known method for a permanent cure for ALS using conventional medicine [27]. Thus, patients with ALS may attempt to visit a multidisciplinary clinic or use complementary and alternative medicine [28, 29].

Prior studies have shown that Mecasin, a herbal extract, has neuro-protective and anti-neuroinflammatory effects, and its safety has been proven in both in vitro and in vivo trials [8–14]; it has been shown to reduce the progression of ALS in Korean Medicine Clinics [6, 7]. No previous randomized controlled trial has examined the effects of Mecasin for the treatment of ALS. The planned clinical trial will determine the optimal dosages of Mecasin and will show whether combined Mecasin and riluzole treatment is both effective and safe for patients with ALS Additional file 1.

## Trial status

Recruitment for this trial opened in August 2016 and will close in February 2018. At the time of manuscript submission, the trial was in the recruitment phase.

## Additional file

Additional file 1: SPIRIT 2013 Checklist: Recommended items to address in a clinical trial protocol and related documents*. (DOC 125 kb)
